# UHPLC-HRMS^n^ Analysis Reveals the Dynamic Metabonomic Responses of *Salvia miltiorrhiza* Hairy Roots to Polysaccharide Fraction from *Trichoderma atroviride*

**DOI:** 10.3390/biom9100541

**Published:** 2019-09-27

**Authors:** Qianliang Ming, Xin Dong, Sijia Wu, Bo Zhu, Min Jia, Chengjian Zheng, Khalid Rahman, Ting Han, Luping Qin

**Affiliations:** 1 School of Pharmacy, Zhejiang Chinese Medical University, Hangzhou 310053, China; mql021@163.com (Q.M.); zhubo@zcmu.edu.cn (B.Z.); 2 Department of Pharmacognosy, School of Pharmacy, Army Medical University, 30 Gaotanyan Street, Chongqing 400038, China; 3 School of Pharmacy, Second Military Medical University, 325 Guohe Road, Shanghai 200433, China; dongxinsmmu@126.com (X.D.); 15868170020@163.com (S.W.); jm71@163.com (M.J.); zheng_chengjian@hotmail.com (C.Z.); 4 Institute of translational medicine, Shanghai University, Shanghai 200444, China; 5 Faculty of Science, School of Pharmacy and Biomolecular Sciences, Liverpool John Moores University, Byrom Street, Liverpool L3 3AF, UK; K.Rahman@ljmu.ac.uk

**Keywords:** *Salvia miltiorrhiza*, hairy roots, *Trichoderma atroviride*, metabolomics, polysaccharide, UHPLC-HRMS^n^

## Abstract

We have previously reported that *Trichoderma atroviride*, an endophytic fungus isolated from *S. miltiorrhiza*, promotes *S. miltiorrhiza* hairy root growth and significantly stimulates the biosynthesis of tanshinones specifically the polysaccharide fraction (PSF). However, this study only focused exclusively on six metabolites whilst ignoring changes to the whole metabolite composition of the *S. miltiorrhiza* hairy roots. In the present study, the dynamic metabonomic responses of *S. miltiorrhiza* hairy roots were investigated using ultra-high-performance liquid chromatography-high resolution mass spectrometry (UHPLC-HRMS^n^). UHPLC-HRMS typical total ions chromatograms (TICs) of PSF-treated hairy root samples were different from the control. Moreover, the results of principal component analysis (PCA), partial least squares discriminant analysis (PLS-DA) and hierarchical clustering analysis (HCA) indicated that PSF-treated samples were significantly different from the control. Through the analysis of PLS-DA, a total of 114 and 99 differential metabolites were found from the positive and negative models respectively and a total of 33 differential metabolites were identified. Thus, *S. miltiorrhiza* hairy roots had been induced to regulate the metabolic profiling in response to PSF and the changes of the metabolic profiling contributed to promoting the biosynthesis of tanshinones notably whilst the biosynthesis of phenolic acids were slightly inhibited.

## 1. Introduction

Endophytic fungi are one kind of microorganisms which can infest the internal tissues of living plants without causing any disease symptoms [1] and are distinguished from pathogens which can lead to disease and reduce the fitness of their host plants [2]. Fossil records indicate that endophytic fungi have been associated with plants since at least 400 million years [3]. In addition, both field and laboratory studies have demonstrated that at least some plant species in natural habitats require endophytic fungi for stress tolerance and survival [4]. The fitness benefits conferred by endophytic fungi are that the fungal endophytes can produce a number of bioactive products themselves and/or promote the biosynthesis of some kind of host plant secondary metabolites which can help their host plants to resist external biotic and abiotic stresses [5,6].

*Salvia miltiorrhiza* Bunge (Labiatae) is an important and well-known traditional Chinese medicinal plant. Its rhizomes called Danshen in China, contain two groups of biologically active compounds: caffeic acid-derived phenolic acids and various tanshinones belonging to diterpene quinones, and are widely used for the treatment of menstrual disorders and cardiovascular diseases and as well for the prevention of inflammation [7]. *S. miltiorrhiza* hairy root is a kind of transformed root induced by infecting the wounded tissue of *S. miltiorrhiza* with the soil bacterium *Agrobacterium rhizogenes* [8]. These roots can produce many similar levels of metabolites comparable to those observed in the whole plant, with the advantages of fast growth rates (in hormone-free media), genetic and biochemical stability. Therefore, *S. miltiorrhiza* hairy root has been established as a useful platform for the metabolic engineering researches superseding the whole plant and a potential means for the production of phenolic acids and tanshinones [9,10]. In a previous study, an endophytic fungus isolated from the root of *S. miltiorrhiza* and identified as *Trichoderma atroviride,* was capable of indigenously producing tanshinone I and tanshinone IIA in rich mycological medium under shake flask conditions [11]. Furthermore, it was observed that the polysaccharide fraction (PSF) isolated from *T. atroviride* can promote hairy root growth and stimulate the biosynthesis of tanshinones in *S. miltiorrhiza* hairy roots [12]. However, this study only focused exclusively on the six metabolites including rosmarinic acid, salvianolic acid B, dihydrotanshinone I, tanshinone I, cryptotanshinone and tanshinone IIA without considering the changes of the whole metabolite compositions in the *S. miltiorrhiza* hairy roots. Therefore, studies of the overall effects of PSF on the *S. miltiorrhiza* hairy roots are needed to improve the understanding of the physiological status and of the metabolic mechanisms associated with *T. atroviride* in the roots of *S. miltiorrhiza*.

Metabolomics is emerging as a valuable technology for the comprehensive profiling and comparison of metabolites which are the end products of cellular regulatory processes, and their levels can be regarded as the ultimate response of biological systems to the alterations of endogenous and/or exogenous factors [13]. It generally consists of global metabolic profiling and multivariate statistical analysis, and thereby provides efficient visualization and identification of the metabolites that depend on plant physiological conditions [14]. Therefore, a global understanding of whole-plant metabolic changes to endogenous and/or exogenous factors is accessible via diverse metabolomic platforms. Currently, chromatography-mass spectrometry (LC-MS and GC-MS) is one of the major methods for detecting and quantifying plant metabolite compositions used in metabolomics [15]. The LC-MS method is a useful tool for analysis of components in herbal medicine [16], and is metabolite-selective and sensitive for the targeted analysis enabling detection of low-concentration compounds which may not be detectable with other methods [17]. Among the LC-MS methods, ultra-high-performance liquid chromatography-mass spectrometry (UHPLC–MS) is a promising tool for investigating the diversity of plant metabolites in metabolomic researches. The reversed phase (RP) chromatography is well established and widely accepted in the field of metabolomics for plant secondary metabolites as it is reliable, robust, repeatable, and the separation mechanisms that cover a very wide range of molecular structures are clearly defined [18].

In the present study, we investigated the dynamic metabonomic responses of *S. miltiorrhiza* hairy roots to PSF from endophytic fungus *T. atroviride* via ultra-high-performance liquid chromatography-high resolution mass spectrometry (UHPLC-HRMS^n^). The aim of this study was to reveal the dynamic metabonomic changes of *S. miltiorrhiza* hairy roots in response to PSF from endophytic fungus *T. atroviride*.

## 2. Materials and Methods 

### 2.1. Chemicals

LC-grade methanol and acetonitrile were brought from Merck (Darmstadt, Germany) and the water used for HPLC was obtained from a Milli-Q system (Millipore, Bedford, MA, USA). Formic acid was obtained from Fluka (Buchs, Switzerland).

### 2.2. Hairy Root Culture 

The *S. miltiorrhiza* hairy roots were derived after infecting the plantlets with a Ri T-DNA bearing *Agrobacterium rhizogenes* bacterium (C58C1). Stock cultures of the hairy roots were maintained on solid, hormone-free half-strength B5 medium with 7.5 g L^−1^ agar and 20 g L^−1^ sucrose, at 25 °C in the dark. All experiments were carried out in shake-flask cultures with 250-mL Erlenmeyer flasks on an orbital shaker set at 180 rpm, at 25 °C with each flask containing 100 mL liquid half-strength B5 medium and inoculated with 1.0 g fresh weight of roots from 3-week-old shake-flask cultures.

### 2.3. Polysaccharide Fraction Preparation and Induction 

The endophytic fungus *T. atroviride* D16 was isolated from the root of *S. miltiorrhiza* [11] and the polysaccharide fraction (PSF) was prepared as previously reported [12]. PSF was then added to the liquid half-strength B5 medium of 3-week-old shake-flask cultured hairy roots at concentrations of 100 mg L^−1^. Control treatments were also added to fresh liquid half-strength B5 medium and the hairy roots were collected at various time intervals (0, 3, 6, 12, 24 days). The hairy roots were harvested from the shake-flasks by filtration and washed three times with distilled water, blotted dried with paper towels, and then grounded into powder in liquid nitrogen and lyophilized in a freeze dryer until a constant dry weight was obtained.

### 2.4. Sample Extraction Procedures

The dry *S. miltiorrhiza* hairy root powders were extracted with methanol (30 mg mL^−1^) under sonication for 60 min. The methanol extract was applied to the UHPLC-MS system for the analysis of the primary and secondary metabolites. 

### 2.5. UHPLC-MS/MS Measurements

UHPLC-MS analysis with high mass accuracy was performed on Agilent 1290 Infinity LC system equipped with Agilent 6520 Accurate-Mass Quadrupole Time-of-Flight (Q-TOF) mass spectrometer (Agilent, Palo Alto, CA, USA). Chromatographic separation was achieved on an ACQUITY UPLC HSS T3 column (2.1 mm × 100 mm, 1.8 μm, Waters, Milford, MA) at 40 °C. The mobile phase consisted of 0.1% formic acid in water (A) and ACN (B), and was delivered at a flow rate of 0.4 mL/min. The following gradient conditions were used: 5% B at 0–2 min and increased to 95% B at 2–17 min, then maintained strong elution for 2 min and followed by equilibrating step of 6 min. The injection volume was 4 μL and the auto-sampler was maintained at 4 °C.

An electrospray ionization source (ESI) was used both in positive mode and negative mode. The optimized conditions were as follows: Capillary voltage, 4 kV for positive mode and 3.5 kV for negative mode; drying gas flow, 11 L/min; gas temperature, 350 °C; nebulizer pressure, 45 psig; fragmentor voltage, 120 V; skimmer voltage, 60 V. Data were collected in centroid mode from 50 to 1100 *m*/*z*. Potential biomarkers were further analyzed by MS/MS, the collision energy was set from 10 to 50 eV.

### 2.6. Data Reduction and Multivariate Data Analysis

The samples were represented by corresponding total ions chromatograms (TIC). The UHPLC–MS raw data were converted to mzXmL format using the Agilent MassHunter Qualitative software. The program XCMS (http://metlin.scripps.edu/download) was used for peak detection, RT alignment and peak integration to generate a visual data matrix. Each column vector contains the quantities of selected metabolites and each row vector describes the abundance of a respective metabolite ion over the entire set of analyses. Each column was normalized to its total spectral count. The data of each sample were imported to SIMCA-P software (Umetrics, Sweden) for principal component analysis (PCA), partial least squares discriminant analysis (PLS-DA), and hierarchical cluster analysis (HCA) to explore correlations between control and PSF treated hairy roots at 3, 6, 12, 18, and 24 d post-elicitation. Statistically significant differences in mean values were tested by student T test for comparisons of two groups by SPSS 19.0. The differences were considered significant when *p* < 0.05.

## 3. Results

### 3.1. Global Metabolite Profiling of the Elicitation Response

**Figure 1:**
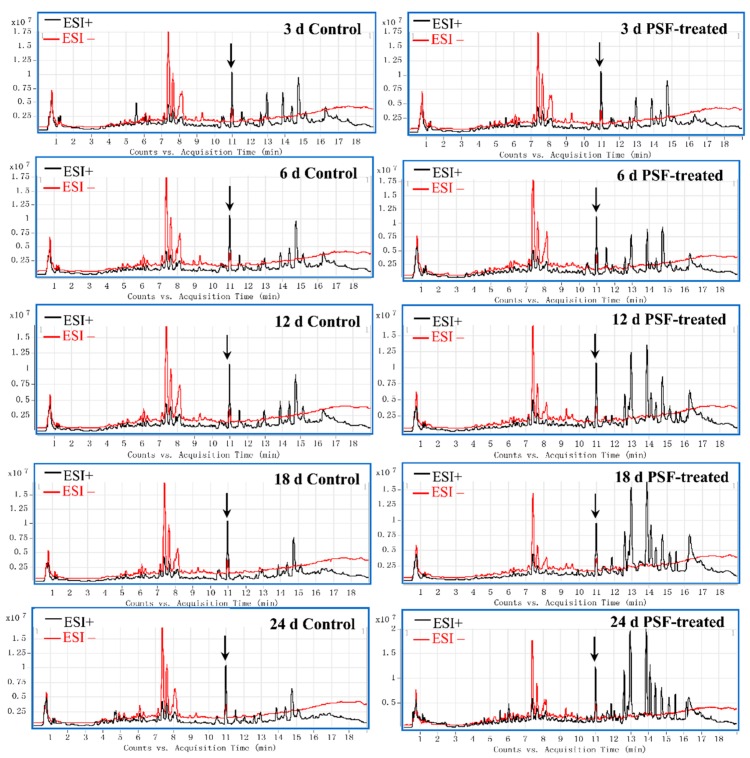
Typical ultra-high-performance liquid chromatography-high resolution mass spectrometry (UHPLC-HRMS) total ions chromatograms (TICs) of methanol extracts from the control and polysaccharide fraction (PSF)-treated *S. miltiorrhiza* hairy roots collected at 3, 6, 12, 18, and 24 d post-elicitation. ESI^+^: black line; ESI^−^: red line. The peak indicated by the arrow was casticin (C_19_H_18_O_8_) added into the extraction solvent methanol as an internal standard.

Methanol extracts of control and PSF-treated *S. miltiorrhiza* hairy roots were analyzed by UHPLC-HRMS. Figure 1 shows typical total ions chromatograms (TICs) obtained at 3, 6, 12, 18, and 24 d after PSF treatment. The peak indicated by the arrow was casticin (C_19_H_18_O_8_) added to the extraction solvent methanol as an internal standard. The most substantial differences in the TICs occurred from at day 3 post PSF treatment in the retention times of 12–16 min at the positive model. Furthermore, as the treatment time prolonged, the differences between control and PSF treatment were markedly different.

### 3.2. Modelling for Metabolic Discrimination

**Figure 2:**
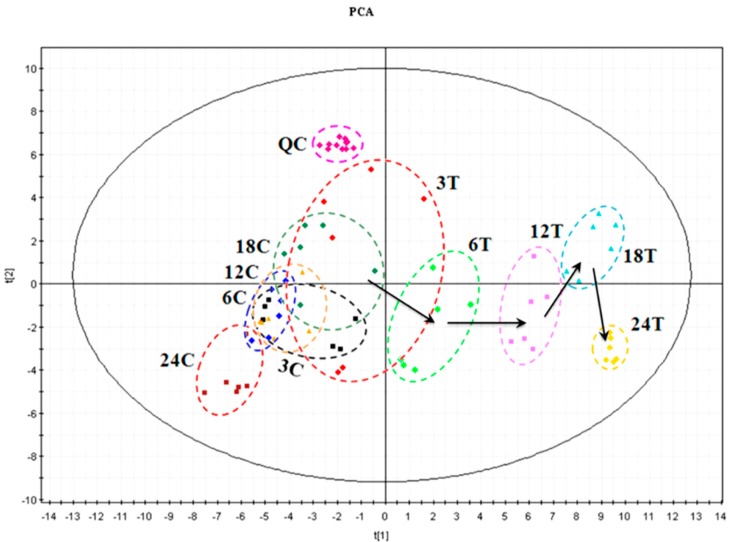
Principal component analysis (PCA) scores plot derived from UHPLC-MS data both in positive mode and negative mode for extracts obtained from the control (C) and PSF-treated (T) *S. miltiorrhiza* hairy roots collected 3, 6, 12, 18 and 24 d post-elicitation. 3C: black squares, 6C: blue rhombus, 12C: yellow triangles, 18C: olive rhombus, 24C: wine squares; 3T: red rhombus, 6T: green rhombus, 12T: pink squares, 18T: cyan triangles, 24T: yellow rhombus.

After aligning mass ions using XCMS, 1399 and 1055 ion peaks were obtained from positive and negative modes respectively. After combining the ion peaks from both positive and negative modes, principal component analysis (PCA) was used to explore correlations between control and PSF treated hairy roots at 3, 6, 12, 18, and 24 d post-elicitation. The PCA scores plot (Figure 2) shows notable and dynamic changes in the metabolone of *S. miltiorrhiza* hairy roots in response to PSF treatment that are not apparent in the control. The samples of control and PSF treatment were different when collected at the time points of 6, 12, 18, and 24 d whilst no differences were observed in the control samples collected at the time points of 3 d. Furthermore, PSF treated samples collected at 6, 12, 18, and 24 d differed significantly with increasing divergence. The supervised method, partial least squares discriminant analysis (PLS-DA), was also applied to investigate the metabolone differences of *S. miltiorrhiza* hairy roots after treatment with PSF. The calculated *R*^2^ values of PCA and *Q*^2^ values of PLS-DA are shown in Table 1 and the values indicate that there were statistically significant difference between the PSF-treated and control samples.

**Figure 3:**
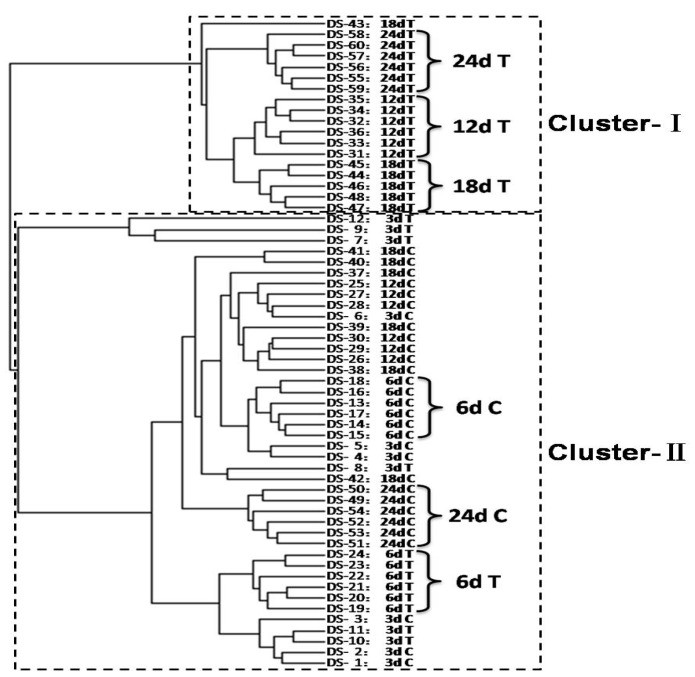
Hierarchical cluster analysis (HCA) dendrogram derived from UHPLC-HRMS data both in positive and negative modes for extracts obtained from the control (C) and PSF-treated (T) *S. miltiorrhiza* hairy roots collected 3, 6, 12, 18, and 24 d post-elicitation.

To visually explore the metabolone differences between the PSF-treated and controlled hairy roots, hierarchical cluster analysis (HCA) was used to analyze all the samples collected at 3, 6, 12, 18, and 24 d. As shown in Figure 3, the 60 *S. miltiorrhiza* hairy root samples were merged into two clusters. Cluster-I includes all the PSF-treated samples collected at 12, 18 and 24 d and it is interesting to note that almost the PSF-treated samples collected at the same time points were merged into small clusters suggesting good reproducibility in the extraction and UHPLC-HRMS measurements. Cluster II contains all the controlled samples collected at 3, 6, 12, 18, and 24 d and the PSF-treated samples collected at 3, 6 d. Furthermore, all PFF-treated samples collected on the 6^th^ d were merged into a small cluster in the cluster-II. The results show that the HCA method can separate the PSF-treated hairy roots from the control at 6, 12, 18, and 24 d. This indicates that the metabolone of PSF-treated hairy roots were dynamic changed and notably different from the control at 6, 12, 18, and 24 d.

### 3.3. Screening and Identification of the Differential Metabolites

**Figure 4:**
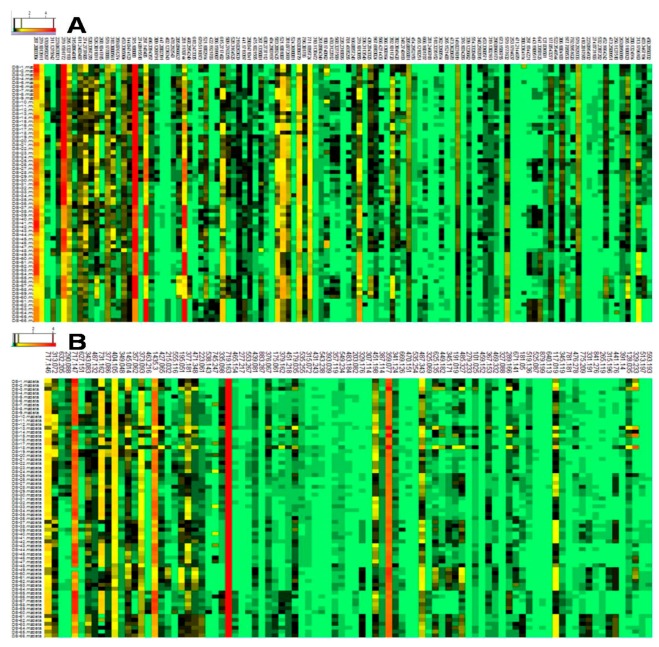
Heatmap analysis of the differential metabolites between the control and PSF treated *S. miltiorrhiza* hairy roots in positive (**A**) and negative (**B**) ion model of UHPLC-HRMS (axis of abscissas indicates the differential metabolites, axis of ordinates indicates the serial number of sample; red indicates high concentration levels of metabolites, yellow and black indicate medium concentration levels of metabolites, green indicates low concentration levels of metabolites).

The variable importance of the projection (VIP) generated after PLS-DA processing was applied to screen the differential metabolites between the PSF-treated and controlled samples at the same time point. A metabolite with a VIP greater than 1.5 was considered to be statistically significantly different. According to the VIP value, a total of 114 and 99 metabolites were conclusively selected as differential metabolites from the positive and negative models respectively. The ionic strengths of these differential metabolites are shown in Figure 4.

The accurate mass is a powerful tool to identify the charged analytes. In our study, the samples were analyzed with the high-resolution mass spectrometer and the exact masses were compared with reference compounds and the *S. miltiorrhiza* chemical composition in-house library for compound identification. To confirm the identification further, the characteristic ions were selected to be MS/MS analyzed with a fragmentor voltage at 200 V and were compared with the references and the network databases. Finally, 33 differential metabolites including 10 tanshinones, 6 phenolic acids, 6 organic acids, 4 amino acids, and 7 other compounds were identified (Table 2).

### 3.4. Metabolite-Specific Profiling

**Figure 5:**
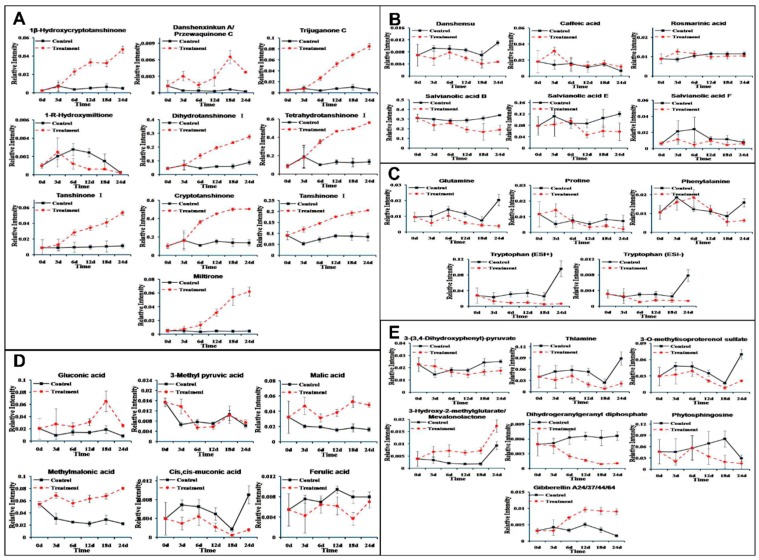
The relative quantification of the identified differential metabolites (**A**) tanshinones; (**B**) phenolic acids; (**C**) amino acids; (**D**) organic acids; and (**E**) other compounds, between the control (black solid lines) and PSF-treated (red dotted lines) *S. miltiorrhiza* hairy roots at different time points post-elicitation.

In order to relatively quantify the metabolites, the abundance of a respective metabolite ion was normalized to its total spectral count. The profiles of the 33 differential metabolites identified between control and PSF-treated hairy roots over the 24-d time course is shown in Figure 5. All the relative quantification of the identified tanshinones was strikingly increased except 1-R-hydroxymiltione (Figure 5A). Among the 6 phenolic acids (Figure 5B), danshensu, salvianolic acid B, and salvianolic acid E were observed to show a downward trend, otherwise the relative quantification of caffeic acid, rosmarinic acid, and salvianolic acid E was significantly different only at some time points. Four amino acids were identified as differential metabolites (Figure 5C). Tryptophan was measured both in the positive and negative models and the changed trends with PSF-treated were basically the same when compared to each other. Tryptophan and glutamine were significantly decreased, while proline and phenytalaine were only significantly different at some time points. Among the 6 organic acids (Figure 5D), gluconic acid, malic acid, and methylmalonic acid were significantly enhanced, and cis, cis-muconic acid and ferulic acid were notably decreased, and 3-methyl pyruvic acid was significantly different only at 3 and 6 d. Among the 7 organic acids (Figure 5E), 3-hydroxy-2-methylglutarate/mevalonolactone and gibberellin A24/37/44/64 were notably increased, and thlamine, 3-*O*-methylisoproterenol sulfate and dihydrogeranylgeranyl diphosphate were significantly decreased, and 3-(3, 4-dihydroxyphenyl)-pyruvate and phytosphingosine were significantly different only at some time points.

## 4. Discussion

We have previously reported that *T. atroviride*, an endophytic fungus isolated from the root of *S. miltiorrhiza*, produces tanshinone I and tanshinone IIA [11]. Furthermore, PSF isolated from the mycelium extract of *T. atroviride* was found to promote *S. miltiorrhiza* hairy root growth and significantly stimulate the biosynthesis of tanshinones in hairy roots [12]. The previous study only analyzed six metabolites including rosmarinic acid, salvianolic acid B, dihydrotanshinone I, tanshinone I, cryptotanshinone, and tanshinone IIA. The present study focuses on the changes to the whole metabolite compositions in the *S. miltiorrhiza* hairy roots and the results show that the metabonomic profiles of the *S. miltiorrhiza* hairy roots were significantly changed after treatment with PSF isolated from *S. miltiorrhiza* endophytic fungus *T. atroviride*. Typical UHPLC-HRMS TICs of PSF-treated hairy root samples were markedly different from the control and the differences between control and treated samples became more and more apparent over the time of PSF-treatment (Figure 1). Moreover, the results of PCA, PLS-DA, and HCA analysis indicated that PSF-treated samples were significantly different from the control (Figure 2, Figure 3, and Table 1). Therefore, *S. miltiorrhiza* hairy roots had been induced to regulate the metabolic profiling in response to PSF. Through the analysis of PLS-DA, a total of 114 and 99 differential metabolites were found from the positive and negative models respectively (Figure 4). In total, 33 differential metabolites including 10 tanshinones, 6 phenolic acids, 6 organic acids, 4 amino acids, and 7 other compounds were identified via comparing with *S. miltiorrhiza* chemical composition in-house library based on the high resolution masses and further conforming with the references and the network databases based on the characteristic ions (Table 2). With the relative quantitative analysis of the identified differential metabolites (Figure 5), it can be concluded that the change to the *S. miltiorrhiza* hairy root metabolic profiling was as a result of notable promotion of the biosynthesis of tanshinones and slight inhibition of the biosynthesis of phenolic acids.

**Figure 6:**
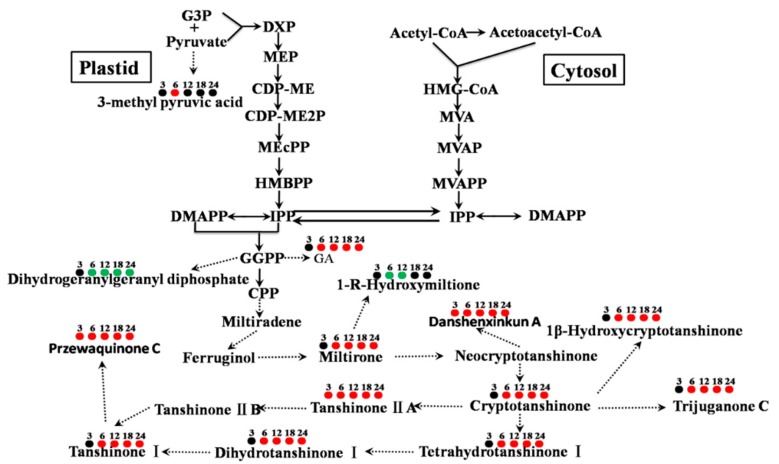
Changes of metabolites associated with tanshinones biosynthesis in *S. miltiorrhiza* hairy roots with PSF treatment. The circles associated with the metabolites indicate whether the metabolite was up-regulation (red), down-regulation (green), or no differences (black) and the numbers above the circles mean the days after PSF treatment.

More than 40 tanshinones, which are a group of abietanoid diterpenes, were isolated from *S. miltiorrhiza* roots [19] and these display cardiovascular and cerebrovascular [20], anti-inflammatory [21], antimicrobial [22], anti-oxidant [23], antitumor [19,24], antiosteoporotic effects [25] besides there medicinal properties. In our study, the relative contents of dihydrotanshinone I, tanshinone I, cryptotanshinone and tanshinone II A (Figure 5A) were dramatically increased in the PSF treated *S. miltiorrhiza* hairy roots confirming our previous study [12]. Moreover, as the intermediate metabolites of tanshinone biosynthesis, miltirone, tetrahydrotanshinone I and 1β-hydroxycryptotanshinone (Figure 5A) were also pronouncedly enhanced. However, the relative contents of 1-R-hydroxymiltione (Figure 5A) and dihydrogeranylgeranyl diphosphates (Figure 5E) were significantly decreased. This was likely due to 1-R-hydroxymiltione and dihydrogeranylgeranyl diphosphates being catalyzed to the downstream metabolites quickly and indicates that the conversions of the two metabolites were not the limited steps of tanshinone biosynthesis (Figure 6). Through the global analysis of *S. miltiorrhiza* hairy root metabolic changes, it can be concluded that in our study the biosynthesis of tanshinones were significantly activated (Figure 6).

More than 50 phenolic acids, a group of caffeic acid derivatives, were isolated from *S. miltiorrhiza* roots [7] and these exert many biological activities including antioxidant, anti-ischemia reperfusion, anti-thrombosis, antihypertension, anti-fibrosis, antivirus, and antitumor properties [26]. In our study, the relative contents of danshensu, salvianolic acid B, salvianolic acid E, and salvianolic F were decreased slightly in the PSF treated *S. miltiorrhiza* hairy roots, whereas caffeic acid and rosmarinic acid were only significantly increased at 3 d (Figure 5B). The changes of rosmarinic acid and salvianolic acid B were a somewhat different when compared to our previous study [12] and could be due to the way samples were dried and detected in the present study.

As we know, tanshinones had much stronger antimicrobial activity than phenolic acids [22], which can explain why the change to the *S. miltiorrhiza* hairy root metabolic profiling was as a result of notable promotion of the biosynthesis of tanshinones whilst slight inhibiting the biosynthesis of phenolic acids. We think that PSF from *T. atroviride* had induced the *S. miltiorrhiza* hairy roots to elicit chemical defense responses for antimicrobial, so the tanshinones, as more efficient antimicrobials, were significantly biosynthesized.

## 5. Conclusions

In the present study, the dynamic metabonomic responses of *S. miltiorrhiza* hairy roots to PSF isolated from endophytic fungus *T. atroviride* via UHPLC-HRMS^n^ were investigated and the results suggest that the hairy roots had been induced to regulate the metabolic profiling in response to PSF. It has been further conferred that PSF had induced the *S. miltiorrhiza* hairy roots to elicit chemical defence responses. Furthermore, with global analysis of the whole metabolite compositions in the *S. miltiorrhiza* hairy roots, it can be concluded that the changes of the metabolic profiling contributed notably to promoting the biosynthesis of tanshinones whilst slightly inhibiting the biosynthesis of phenolic acids. Hence, we conclude that PSF can be considered as a potent elicitor for the large-scale production of tanshinones in *S. miltiorrhiza* hairy root culture systems and can be used as a tool to investigate the biosynthetic pathway of tanshinones.

## Figures and Tables

**Table 1:** Parameters of PCA and partial least squares discriminant analysis (PLS-DA) models by the control and treated models.

Time Point	Ion Model	Type	A	R^2^X	R^2^Y	Q^2^
3 d	ESI+	PCA	2	0.658		0.495
		PLS-DA	4	0.761	0.991	0.864
	ESI−	PCA	2	0.825		0.501
		PLS-DA	2	0.777	0.593	0.239
6 d	ESI+	PCA	2	0.695		0.557
		PLS-DA	2	0.675	0.991	0.959
	ESI−	PCA	2	0.655		0.470
		PLS-DA	2	0.525	0.997	0.951
12 d	ESI+	PCA	2	0.788		0.683
		PLS-DA	2	0.744	0.996	0.974
	ESI−	PCA	2	0.766		0.645
		PLS-DA	2	0.760	0.992	0.981
18d	ESI+	PCA	3	0.880		0.635
		PLS-DA	2	0.755	0.993	0.958
	ESI−	PCA	3	0.820		0.635
		PLS-DA	2	0.68	0.994	0.923
24 d	ESI+	PCA	2	0.846		0.729
		PLS-DA	2	0.834	0.999	0.994
	ESI−	PCA	2	0.819		0.682
		PLS-DA	2	0.796	0.999	0.994
3–24 d	ESI+	PCA	12	0.889		0.759
		PLS-DA	19	0.925	0.941	0.627
	ESI−	PCA	10	0.879		0.757
		PLS-DA	13	0.897	0.966	0.79
3–24 d	ESI±	PCA	11	0.876		0.771
		PLS-DA	18	0.912	0.953	0.654

**Table 2:** The list of identified differential metabolites and their characteristic fragment ions.

NO.	RT(min)	mz	Ion	Formula	Identification	Fragments
***Tanshinones***						
1 ^△^	9.88	313.143	[M + H]^+^	C_19_H_20_O_4_	1β-Hydroxycryptotanshinone	313.1432, 295.1315, 277.1233, 267.1360, 253.1219, 249.1264, 225.0903, 209.0952, 183.0796, 165.0690, 141.0688
2 ^●^	11.74	295.098	[M − H]^−^	C_18_H_16_O_4_	Danshenxinkun A/Przewaquinone C	295.0985, 279.0658, 267.1064, 249.0924, 237.0926, 222.0690, 209.0628, 183.0086, 165.0712, 148.4306
3 ^△^	11.80	341.138	[M + H]^+^	C_20_H_20_O_5_	Trijuganone C	341.1380, 281.1165, 263.1069, 253.1197, 235.1113, 220.0881, 207.1166, 192.0923, 179.0850, 169.0639, 153.0697, 145.1000, 135.1176, 124.0449, 107.0836
4 ^△^	12.47	343.155	[M − H]^−^	C_19_H_22_O_3_	1-R-Hydroxymiltione	343.1603, 299.1671, 284.1423, 256.1112, 243.1040, 228.0799, 216.0804, 199.0765, 186.0690, 173.0253, 160.0522, 127.6810, 106.5153
5 *	12.55	279.101	[M + H]^+^	C_18_H_14_O_3_	Dihydrotanshinone I	279.1020, 261.0910, 251.1060, 233.0960, 218.0730, 209.0960, 205.1010, 190.0770, 169.0650
6 ^△^	12.91	281.117	[M + H]^+^	C_18_H_16_O_3_	Tetrahydrotanshinone 1	281.1172, 263.1067, 248.0825, 235.1106, 220.0880, 207.1165, 192.0931, 179.0850, 169.0645, 153.0701, 141.0698, 115.0554
7 *	13.78	277.086	[M + H]^+^	C_18_H_12_O_3_	Tanshinone I	277.0860, 262.0620, 249.0910, 234.0670, 221.0960, 203.0850, 193.1010, 178.0770, 169.0650
8 *	13.81	297.148	[M + H]^+^	C_19_H_20_O_3_	Cryptotanshinone	297.1490, 282.1250, 279.1380, 268.1090, 254.0940, 251.1430, 236.1187, 221.0954, 209.0958, 193.1014, 181.1004, 165.0698, 143.0495
9 *	15.10	295.133	[M + H]^+^	C_19_H_18_O_3_	Tanshinone IIA	295.1330, 280.1090, 277.1230, 266.0940, 262.0990, 252.0780, 249.1270, 235.0750, 221.1320, 207.0810
10 ^△^	15.45	283.169	[M + H]^+^	C_19_H_22_O_2_	Miltirone	283.1710, 268.1492, 253.1211, 240.1145, 225.0914, 207.0813, 195.1163, 180.0922, 165.0698, 153.0711, 139.0041
***Phenolic acids***						
11 *	4.07	197.045	[M − H]^−^	C_9_H_10_O_5_	Danshensu	197.0463, 179.0353, 135.0453, 123.0452, 109.0292, 89.0402, 72.9930, 67.0192, 53.0392
12 *	5.61	179.035	[M − H]^−^	C_9_H_8_O_4_	Caffeic acid	179.0353, 135.0445, 117.0340, 108.0218, 89.0398, 79.0556, 65.0038
13 *	7.34	359.078	[M − H]^−^	C_18_H_16_O_8_	Rosmarinic acid	359.0780, 197.0460, 179.0350, 161.0240, 135.0450, 72.9930
14 *	7.78	717.147	[M − H]^−^	C_36_H_30_O_16_	Salvianolic acid B	717.1480,519.0950,321.0420
15 ^△^	8.21	717.147	[M − H]^−^	C_36_H_30_O_16_	Salvianolic acid E	717.1480, 519.0950, 339.0530, 321.0420
16 ^△^	9.24	315.086	[M + H]^+^	C_17_H_14_O_6_	Salvianolic acid F	163.0390, 145.0285, 135.0440, 117.0335, 107.0493, 89.0387
***Amino acids***						
17	0.70	147.076	[M + H]^+^	C_5_H_10_N_2_O_3_	Glutamine	147.0766, 130.0501, 102.0551, 84.0449, 56.0496
18	2.50	116.071	[M + H]^+^	C_5_H_9_NO_4_	Proline	116.0707, 99.0437, 84.0442, 71.0494, 56.0494
19 ^#^	3.54	166.086	[M + H]^+^	C_9_H_11_NO_2_	Phenylalanine	166.0861, 149.0601, 131.0486, 120.0807, 103.0540, 84.9599, 79.0530
20 ^#^	4.71	205.097	[M + H]^+^	C_11_H_12_N_2_O_2_	Tryptophan	205.0966, 188.0700, 159.0917, 146.0599, 132.0805, 118.0650
	4.71	203.082	[M − H]^−^			203.0382, 186.0552, 159.0930, 142.0665, 116.0510, 74.0253
***Organic acids***						
21 ^#^	0.71	195.051	[M − H]^−^	C_6_H_12_O_7_	Gluconic acid	195.0512, 177.0394, 159.0294, 129.0194, 99.0288, 75.0092, 59.0139
22 ^#^	0.92	101.025	[M − H]^−^	C_4_H_6_O_3_	3-methyl pyruvic acid	101.0248, 83.0138, 73.0286, 57.0351
23 ^#^	0.96	133.014	[M − H]^−^	C_4_H_6_O_5_	Malic acid	133.0143, 115.0039, 89.0249, 71.0142, 59.0144
24 ^#^	1.42	117.019	[M − H]^−^	C_4_H_6_O_4_	Methylmalonic acid	117.0193, 99.0089, 73.0302, 55.019
25 ^#^	4.09	143.034	[M + Na]^+^	C_6_H_6_O_4_	cis, cis-Muconic acid	143.0341, 126.0342, 113.0291, 97.9684, 69.0337, 55.0181
26	5.17	239.056	[M + FA − H]^−^	C_10_H_10_O_4_	Ferulic acid	197.0457, 179.035, 135.0452
***Other compounds***						
27	0.69	219.026	[M + Na]^+^	C_9_H_8_O_5_	3-(3,4-Dihydroxyphenyl)pyruvate	219.1202, 202.0934, 174.098, 156.0889, 130.0724, 104.0946, 84.0695
28 ^#^	0.72	265.111	[M + H]^+^	C_12_H_16_N_4_OS	Thiamine	265.1114, 248.1132, 144.0479, 122.0716
29	3.57	306.100	[M + H]^+^	C_12_H_19_NO_6_S	3-O-Methylisoproterenol Sulfate	306.0955, 186.0577, 144.0476, 126.037, 113.0291, 99.0261, 85.0284, 69.03367
30 ^●^	5.14	175.061	[M + FA − H]^−^	C_6_H_10_O_3_	3-hydroxy-2-methylglutarate/ Mevalonolactone	175.0612, 157.0512, 131.0721, 115.0403, 85.0664, 59.0148
31	7.90	475.196	[M + Na]^+^	C_20_H_38_O_7_P_2_	Dihydrogeranylgeranyl diphosphate	475.1922, 393.2455, 342.2300, 297.1884, 164.1471, 209.1525, 181.0498, 163.0377, 135.1163, 107.0857, 93.0700, 85.0286, 71.0491, 57.0698
32	10.45	318.300	[M + H]^+^	C_18_H_39_NO_3_	Phytosphingosine	318.3003, 300.2896, 256.2629, 212.2368, 146.1174, 132.1017, 102.0917, 88.0753, 70.0652, 57.0702
33 ^●^	11.26	347.185	[M + H]^+^	C_20_H_26_O_5_	Gibberellin A44/24/37/64	347.1850, 329.1753, 301.1793, 283.1688, 233.0808, 187.0736, 169.0630, 163.0761, 141.0692, 121.1000, 95.0854, 55.0540

* indicates that the metabolite is identified by compared with the reference and the MS/MS spectra of these metabolites are shown in Supplementary Materials Figure S1. # indicates that the metabolite is identified by compared with the network databases. ^●^ indicates that the metabolite is identified as one of the isomers basing on the mass data.

## Supplementary Materials

The following are available online at https://www.mdpi.com/2218-273X/9/10/541/s1.
