# Supplementary material for: UHPLC-HRMSn Analysis Reveals the Dynamic Metabonomic Responses of Salvia miltiorrhiza Hairy Roots to Polysaccharide Fraction from Trichoderma atroviride
Source: Biomolecules. 2019 Sep 27;9(10):541. doi: 10.3390/biom9100541 (PMC6843243; doi:10.3390/biom9100541)
Supplement: Supplementary file 1 [file biomolecules-09-00541-s001.pdf]

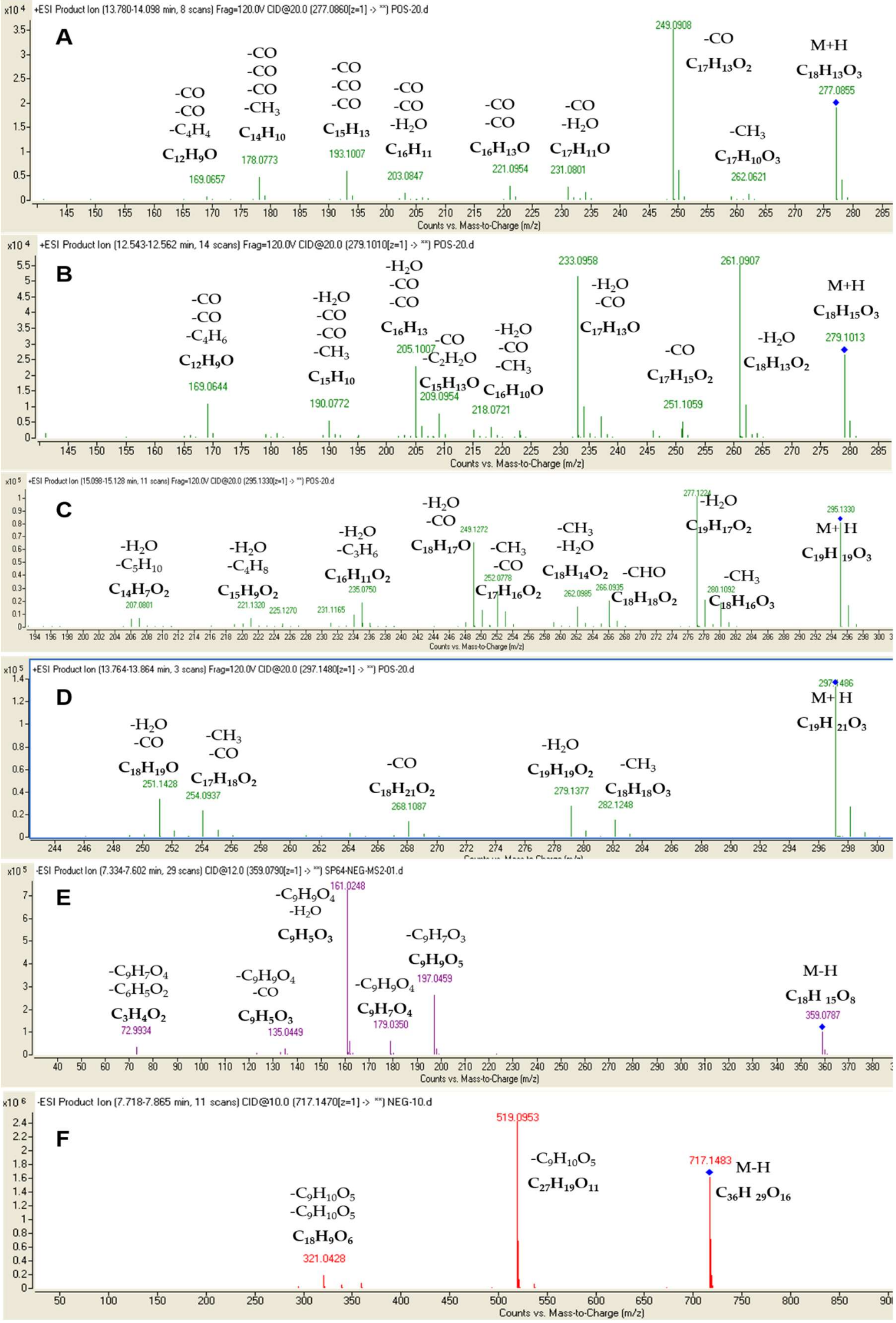

Figure S1 The MS/MS spectra of the metabolites identified by compared with the references (A,Tanshinone Ⅰ; B, Dihydrotanshinone I; C, Tanshinone IIA; D, Cryptotanshinone; E. Rosmarinic acid; F, Salvianolic acid B)
